# Removal

**Published:** 1875-01

**Authors:** 


### REMOVAL.

We have taken, for a term of years, the fine, spacious rooms at No. 137 Eighth street, three doors from Broadway, to which location we shall shortly remove our New York office. We do this for the greater convenience of our patrons, and because our business has greatly outgrown our old quarters at 84 Broadway. Our new store is in the centre of the publishing trade. Directly opposite are the establishments of Hurd & Houghton, Francis, McMillan, Wiley, and the Clinton Hall Auction room of the Leavitts, where the trade sales of books are held. This quarter is known as "Book Row," and here the book-trade is establishing its permanent centre. The "Bible House" and Cooper Institute are in the immediate vicinity, as is also the great Mercantile Library. We shall be able to do our work more promptly and satisfactorily by this increase of our facilities, while those of our customers who visit us in person will appreciate the advantages of the change. Our London office remains, as heretofore, at No. 17 Henrietta street, Covent Garden, from which we supply public and private libraries, in all countries, with our own publications, as well as all choice books and periodicals, wherever published. Letters designed for either the New York or London office should bear the address of "The American & Foreign Publication Company," with the addition of the street and number, as above stated.

---

We are in receipt of complaints of "Hall's Health Publishing Company," 40 Broadway, which sometimes issues a pamphlet called "Hall's *American* Journal of Health." It is trading on our good name, and should not be encouraged.

Hard and Soft Water.—There is a notion quite prevalent in the minds of the people that the drinking of hard water is injurious to health, and most physicians have warned mankind to as far as possible avoid the practice. But the truth is that hard water is not only clearer, colder, more free from air and more agreeable to the taste than soft, but that it is less liable to the absorption of organic matter and to the sustenance of the life of zymotic organisms, or to exert solvent properties upon salts of iron or upon leaden conducting pipes. It is said that the lime infused in hard water, exerts a beneficial influence upon the body. A practical test of the truth of this new theory is to be had in the case of the residents of mountainous districts, where the water is almost invariably hard, and where the inhabitants exhibit the best physical development. Water containing about six grains of carbonate of lime to the gallon is suitable for use in all household purposes, for such water offers the necessary amount of carbonate of lime for the support of life in the simplest and most digestible form.

---

The Type Writer.—This remarkable invention, which prints faster than most men can write, is creating a great sensation. Some reporters for the daily press are now practising on the machine, intending to use it in reporting speeches instead of the short-hand system, which they expect to equal in speed, while at the same time producing printed "copy" instead of hieroglyphics to be afterwards written out. The machines can be seen at 751 Broadway.

---

Wash yourself all over daily, occupying not over five minutes in the process, and you will ward off many diseases.

